# Ferimzone (Third Edition) (Pesticides)

**DOI:** 10.14252/foodsafetyfscj.D-25-00007

**Published:** 2025-03-21

**Authors:** Food Safety Commission of Japan

## Abstract

Food Safety Commission of Japan (FSCJ) conducted a risk assessment of ferimzone (CAS No. 89269-64-7), a pyrimidine hydrazone fungicide, based on submitted documents. A request for reevaluation was made under the Agricultural Chemical Regulation Act. Additional information was submitted by the Ministry of Agriculture, Forestry and Fisheries, which included data on residues in crops (paddy rice) and in livestock products (cattle and chickens), fate in livestock (goats and chickens), and also related published scientific literatures. Major adverse effects of ferimzone were observed in the liver (including centrilobular hypertrophy of hepatocytes) and blood (anemia). Adverse effects were observed on neither fertility, teratogenicity, nor genotoxicity. The lowest no-observed-adverse-effect level (NOAEL) obtained from these studies was 1.94 mg/kg bw per day in the two-year combined chronic toxicity/carcinogenicity study in rats. FSCJ specified an acceptable daily intake (ADI) of 0.019 mg/kg bw per day by applying a safety factor of 100 to this NOAEL. The lowest value was a NOAEL of 30 mg/kg bw per day in the general pharmacological study in mice and rats, as well as the one-year chronic toxicity study in dogs. FSCJ specified an acute reference dose (ARfD) of 0.3 mg/kg bw by applying a safety factor of 100 to this NOAEL.

## Conclusion in Brief

Food Safety Commission of Japan (FSCJ) conducted a risk assessment of ferimzone (CAS No. 89269-64-7), a pyrimidine hydrazone fungicide, based on submitted documents. A request for reevaluation was made under the Agricultural Chemical Regulation Act. Additional information was submitted by the Ministry of Agriculture, Forestry and Fisheries, which included data on residues in crops (paddy rice) and in livestock products (cattle and chickens), fate in livestock (goats and chickens), and also related published scientific literatures.

The data used in the assessment include fate in plants (paddy rice), residues in crops, fate in livestock (goats and chickens), residues in livestock products, fate in animals (rats), subacute toxicity (rats and mice), chronic toxicity (dogs), two-year combined chronic toxicity/carcinogenicity (rats), carcinogenicity (mice), two-generation reproductive toxicity (rats), developmental toxicity (rats and rabbits) and genotoxicity.

Major adverse effects of ferimzone were observed in the liver (including centrilobular hypertrophy of hepatocytes) and blood (anemia). Adverse effects were observed on neither fertility, teratogenicity, nor genotoxicity.

Increased incidences of squamous cell carcinoma of the nasal cavity were observed in both male and female rats in the two-year combined chronic toxicity/carcinogenicity study. The mode of action was, however, considered to be non-genotoxic, and it was deemed possible to establish a threshold for evaluation.

Based on these results, relevant substances for the residue definitions for dietary risk assessments were identified as ferimzone (parent compound) and its metabolite B in agricultural and fishery products, and ferimzone (parent compound only) in livestock products.

The lowest no-observed-adverse-effect level (NOAEL) obtained from these studies was 1.94 mg/kg bw per day in the two-year combined chronic toxicity/carcinogenicity study in rats. FSCJ specified an acceptable daily intake (ADI) of 0.019 mg/kg bw per day by applying a safety factor of 100 to this NOAEL.

NOAEL and lowest-observed-adverse-effect level (LOAEL) values across these studies were compared for potential adverse effects of a single oral administration of ferimzone. The lowest value was a NOAEL of 30 mg/kg bw per day in the general pharmacological study in mice and rats, as well as the one-year chronic toxicity study in dogs. FSCJ specified an acute reference dose (ARfD) of 0.3 mg/kg bw by applying a safety factor of 100 to this NOAEL.

**Table 1.** *Levels relevant to toxicological evaluation of ferimzone*

Species	Study	Dose (mg/kg bw per day)	NOAEL (mg/kg bw per day)^1)^	Reference (Dossier for pesticides evaluation)
Rat	90-day subacute toxicity study	0, 250, 1 000, 4 000, 8 000 ppm	M: 16.4 F:1 8.3 M: Increased ALP levels F: Decreases in Ht and Hb levels	M: 16.4 F: 18.3 M: Increased ALP levels F: Decreases in Ht and Hb levels
		M: 0, 16.4, 65.9, 268, 501 F: 0, 18.3, 73.2, 278, 501		
	Two-year combined chronic toxicity/carcinogenicity study	0, 50, 500, 3 000 ppm	M: 1.94 F: 23.0 M/F: Suppressed body weight gain, etc. (Increased incidence of squamous cell carcinoma of the nasal cavity)	M: 1.94 F: 23.0 M/F: Suppressed body weight gain, etc. (Increased incidence of squamous cell carcinoma of the nasal cavity)
		M: 0, 1.94, 19.2, 123 F: 0, 2.26, 23.0, 145		
	Two-generation reproductive toxicity study	0, 200, 600, 1 800 ppm	Parents PM: 45.0 F_1_M: 62.9 PF: 55.5 F_1_F: 66.9 Offspring PM: 15.1 F_1_M: 19.7 PF: 19.3 F_1_F: 21.1 Parents M/F: Suppressed body weight gain, etc. Offspring Low body weight (No effect on fertility is observed)	Parents and offspring PM: 15.1 F_1_M: 19.7 PF: 19.3 F_1_F: 21.1 Parents M: Suppressed body weight gain, etc. F: Increased water intake Offspring Low body weight (No effect on fertility is observed)
		PM: 0, 15.1, 45.0, 136 PF: 0, 19.3, 55.5, 159 F_1_M: 0, 19.7, 62.9, 197 F_1_F: 0, 21.1, 66.9, 202		
	Developmental toxicity study	0, 2, 6, 18, 54	Dams: 18 Fetuses: 54 Dams: Suppressed body weight gain Fetuses: No toxicity (No teratogenicity observed)	Dams: 18 Fetuses: 54 Dams: Suppressed body weight gain Fetuses: No toxicity (No teratogenicity observed)
Mouse	90-day subacute toxicity study	0, 250, 1 000, 4 000, 8 000 ppm	M: 124 F: 143 FM: Suppressed body weight gain, etc.	M: 30.6 F: 143 M: Increased relative weights of the liver F: Suppressed body weight gain, etc.
		M: 0, 30.6, 124, 445, 792 F: 0, 33.3, 143, 521, 910		
	18-month genotoxicity study	0, 50, 500, 3 000 ppm	M: 4.75 F: 5.16 FM: Suppressed body weight gain, etc. (No carcinogenicity is observed)	M: 4.75 F: 5.16 FM: Suppressed body weight gain, etc. (No carcinogenicity is observed)
		M: 0, 4.75, 48.4, 302 F: 0, 5.16, 52.7, 354		
Rabbit	Developmental toxicity study	0, 8, 25, 75	Dams: 25 Fetuses: 8 Dams: Suppressed body weight gain, etc. Fetuses: Increases in post-implantation embryonic and fetal mortalities (No teratogenicity is observed)	Dams: 25 Fetuses: 8 Dams: Suppressed body weight gain, etc. Fetuses: Increases in post-implantation embryonic and fetal mortalities (No teratogenicity is observed)
Dog	One-year chronic toxicity study	0, 10, 30, 100	M: 10 F: 10 F: Suppressed body weight gain, etc. M: Decreased food intake, etc.	M: 10 F: 10 F: Suppressed body weight gain, etc. M: Decreased food intake, etc.
ADI			NOAEL: 1.94 ADI: 0.019 SF: 100	NOAEL: 1.94 ADI: 0.019 SF: 100
The critical study for setting ADI (cRfD)			Two-year combined chronic toxicity /carcinogenicity study (rat)	Two-year combined chronic toxicity/ carcinogenicity study (rat)

ADI, Acceptable daily intake; ALP, Alkaline phosphatase; Ht, Hematocrit; Hb, Hemoglobin; LOAEL, Lowest-observed-adverse-effect level; NOAEL, No-observed-adverse-effect level; SF, Safety factor

^1)^ The adverse effect observed at LOAEL.

-, NOAEL could not be specified.

**Table 2.** *Potential adverse effects of a single oral administration of ferimzone*

Species	Study	Dose (mg/kg bw or mg/kg bw per day)	Endpoints relevant to setting NOAEL and ARfD (mg/kg bw or mg/kg bw per day)^1)^
Rat	Acute toxicity study	0, 296, 385, 500, 650, 845, 1 100, 1 430 (M only)	M/F: - M/F: Decreased locomotor activities, abnormal gait, etc.
Mouse	Acute toxicity study	0, 385, 500, 650, 845, 1 100, 1 430	M/F: - M/F: Decreased locomotor activities, abnormal gait, etc.
	General pharmacological study (general condition)	M: 0, 30, 120, 480	M: 30 M: Ataxia, abnormal gait, etc.
Rabbit	General pharmacological study (general condition)	M: 0, 30, 120, 480	M: 30 M: Decreases in locomotor activities and hopping reaction
Dog	One-year chronic toxicity study	0, 10, 30, 100	M/F: 30 M/F: Suppressed body weight gain
ARfD			NOAEL: 30 ARfD: 0.3 SF: 100
The critical study for setting ARfD			General pharmacological studies (rabbit and dog) and one-year chronic toxicity study (dog)

ARfD, Acute reference dose; NOAEL, No-observed-adverse-effect level; SF, Safety factor

^1)^ The adverse effect observed at LOAEL

-, NOAEL could not be specified.

## References

[1] Food Safety Commission of Japan. Risk Assessment Report. Ferimzone (Third Edition) (Pesticides) [in Japanese]. https://www.fsc.go.jp/fsciis/attachedFile/download?retrievalId=kya20231025171&fileId=211.

